# Dynamic Changes of BVRA Protein Levels Occur in Response to Insulin: A Pilot Study in Humans

**DOI:** 10.3390/ijms24087282

**Published:** 2023-04-14

**Authors:** Flavia Agata Cimini, Antonella Tramutola, Ilaria Barchetta, Valentina Ceccarelli, Elena Gangitano, Simona Lanzillotta, Chiara Lanzillotta, Maria Gisella Cavallo, Eugenio Barone

**Affiliations:** 1 Department of Experimental Medicine, Sapienza University of Rome, 00185 Rome, Italy; 2 Department of Biochemical Sciences “A. Rossi-Fanelli”, Sapienza University of Rome, 00185 Rome, Italy

**Keywords:** biliverdin reductase-A, insulin signaling, metabolism, obesity, diabetes

## Abstract

Biliverdin reductase-A (BVRA) is involved in the regulation of insulin signaling and the maintenance of glucose homeostasis. Previous research showed that BVRA alterations are associated with the aberrant activation of insulin signaling in dysmetabolic conditions. However, whether BVRA protein levels change dynamically within the cells in response to insulin and/or glucose remains an open question. To this aim, we evaluated changes of intracellular BVRA levels in peripheral blood mononuclear cells (PBMC) collected during the oral glucose tolerance test (OGTT) in a group of subjects with different levels of insulin sensitivity. Furthermore, we looked for significant correlations with clinical measures. Our data show that BVRA levels change dynamically during the OGTT in response to insulin, and greater BVRA variations occur in those subjects with lower insulin sensitivity. Changes of BVRA significantly correlate with indexes of increased insulin resistance and insulin secretion (HOMA-IR, HOMA-β, and insulinogenic index). At the multivariate regression analysis, the insulinogenic index independently predicted increased BVRA area under curve (AUC) during the OGTT. This pilot study showed, for the first time, that intracellular BVRA protein levels change in response to insulin during OGTT and are greater in subjects with lower insulin sensitivity, supporting the role of BVR-A in the dynamic regulation of the insulin signaling pathway.

## 1. Introduction

Biliverdin reductase-A (BVRA), mainly known as the enzyme responsible for bilirubin production in the degradation pathway of heme, has recently drawn attention for its role in regulating insulin signaling and participating in the maintenance of metabolic homeostasis [1]. BVRA is endowed with a dual-specificity serine/threonine/tyrosine (Ser/Thr/Tyr) kinase activity [2,3], a scaffold protein function [3,4,5,6,7,8,9], and transcription functions [10] directly involved in the regulation of the cell redox metabolism and of the complex insulin signaling pathway at different levels. Alterations of BVRA protein levels influence many metabolic processes, such as glucose uptake, regulation of lipid and protein metabolism, cell proliferation, differentiation, and death [4,5,6,11,12,13,14,15,16].

Insulin responses are promoted through the phosphorylation (activation) of the insulin receptor substrates-1 and -2 (IRS1 and IRS2) complexes. Such activation is achieved following the binding of insulin to the extracellular domain of the IR, which autophosphorylates on tyrosine (Tyr) residues. Such phosphorylation is required for the activation of IR kinase activity, that in turn promotes IRS1 phosphorylation. The coupling between IR and IRS1 is crucial for the induction of the intracellular cascade. Like IRS1, BVRA is a direct target of IR [11], which phosphorylates both BVRA and IRS1 on specific Tyr residues thus resulting in their activation [11]. Then, as part of a regulatory loop, BVRA phosphorylates IRS1 on inhibitory Ser residues (e.g., Ser307) to avoid IRS1 aberrant activation in response to IR [11]. Once activated, IRS1 works as a scaffold protein, driving the activation of the two main arms of the insulin signaling: (1) the Ras/Raf/MAPK pathway (ERK1/2) mainly involved in gene transcription and (2) the PI3K/Akt axis that is critical for glucose uptake as well as for protein and lipid metabolism [17]. Moreover, Akt promotes the phosphorylation of several targets, among which are (1) GSK3β (on Ser9, inhibitory site), which has a role in energy production, and (2) mTOR (on Ser2448, activating site), which regulates protein synthesis and autophagy [17]. Within both axes, BVRA works as a kinase or as a scaffold protein facilitating (1) ERK1/2 phosphorylation and the subsequent translocation in the nucleus and followed by the activation of Elk1 [4,14]; (2) the PDK1-mediated activation of Akt [6]; (3) PDK1-mediated activation of the atypical PKCζ [5]; and (4) the Akt-mediated inhibition of GSK3β [9]. Moreover, BVRA was found to be essential for the AMPK-mediated inhibition of mTOR [8,18]. Under conditions of energy depletion, AMPK directly senses increases in AMP:ATP and ADP:ATP ratios, thus promoting the inhibition of mTOR to block the processes that deplete cellular ATP (e.g., protein synthesis and cell cycle progression, controlling cell size and preventing apoptosis) [18,19,20]. In addition, by favoring the inhibition of GSK3β, BVRA prevents the degradation of the peroxisome proliferator-activated receptor alpha (PPARα), which has a main role in lipid metabolism [21].

Findings from animal models of obesity showed that hepatic- or adipocyte-specific deletion of BVRA hampers the insulin cascade and is associated with impaired glucose metabolism [21,22,23,24]. In these mice, a high-fat diet regimen leads to hyperinsulinemia but reduced activation of the AKT and MAPK pathways both in the liver and in the adipocytes [21,22,23,24]. Similarly, reduced BVRA protein levels prompted the development of brain insulin resistance in animal models of aging or neurodegenerative diseases [3,25,26]. Conversely, improving BVRA functions ameliorated the insulin signaling activation, by fostering the IR/AKT/GSK3β axis, which resulted in increased glucose uptake in diabetic mice [22] and in the brain [27,28]. 

The results collected in animal models of metabolic disorders were further strengthened by our group in humans. Our recent studies have shown that a consistent reduction in BVRA protein levels is present in individuals with obesity and type-2 diabetes (T2D) [29,30,31]. Reduction in BVRA protein levels was linked to abnormal activation of the IR/IRS1/AKT/GSK3β/GLUT4 pathway, which was related to poor glycometabolic control, as well as the presence of metabolic syndrome, liver steatosis, and inflammation of visceral adipose tissue in individuals with obesity and T2D [29,30,31,32].

While the role of BVRA in regulating the insulin signaling pathway and its negative effects in the event of loss have been well-established, no studies in the literature have investigated whether BVRA protein levels change dynamically within the cells. It is currently unknown if BVRA protein levels follow the same pattern as circulating glucose and insulin levels. The purpose of this study is to examine if intracellular BVRA protein levels change in response to glucose or insulin in humans. The study will use peripheral blood mononuclear cells (PBMCs) isolated from blood samples collected during an oral glucose tolerance test (OGTT) on a group of subjects with different levels of insulin sensitivity to determine the relationship between intracellular BVRA protein levels and circulating glucose and insulin levels. PBMCs are increasingly used as a surrogate model for glucose disposal. Recent studies have shown that PBMCs have insulin-sensitive GLUT4 activity and that GLUT4 increases on the membrane of monocytes [33,34,35] and lymphocytes [36,37] in response to insulin. Furthermore, we previously reported alterations of the insulin signaling cascade in PBMCs from obese individuals [29]. In addition, PBMCs are easy to collect repeatedly in sufficient quantities [38,39,40]. Hence, PBMCs could potentially serve as a proxy tissue [33,35] allowing sampling of a large study population and making the procedures necessary for research less invasive. The use of PBMCs therefore helps to perform cellular-based studies aimed to evaluate changes in response to circulating glucose or insulin levels.

## 2. Results

### 2.1. Dynamic Changes of BVRA Protein Levels during OGTT in Humans

**Figure 1:**
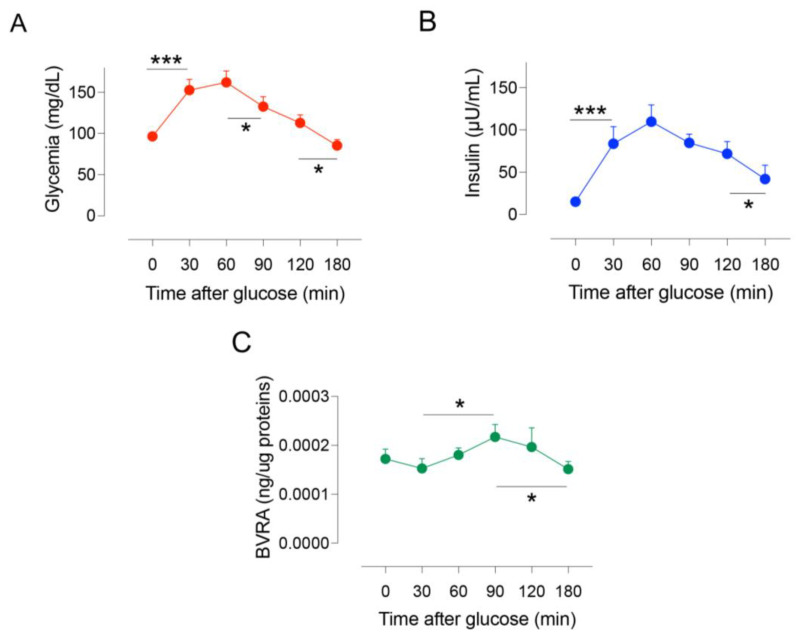
Changes of (**A**) glycemia (mg/dL) and (**B**) insulin (μU/mL) circulating levels along with (**C**) BVRA protein levels evaluated in PBMCs isolated from subjects with different levels of insulin sensitivity (*n* = 12) underwent an oral glucose tolerance test (OGTT). Data are presented as mean ± SEM. * *p* < 0.05, *** *p* < 0.01 (paired Student’s *t*-test).

A significant overall variation across the time (T0–T180) of both glycemia (one-way ANOVA F(5,53) = 17.85, *p* < 0.0001) and insulin circulating levels (one-way ANOVA F(5,54) = 9.138, *p* < 0.0001) under the OGTT, was observed (Figure 1A,B). Glycemia levels show a significant increase at T30, remain elevated at T60, and show the first significant reduction between T90 and T120 (Figure 1A). Unlike glycemia, insulin levels significantly increase at T30 and remain elevated up to T120, reaching comparable levels to those measured at T0 at the end of the test at T180 (Figure 1B).

Changes of BVRA protein levels were evaluated by ELISA in PBMCs isolated during the OGTT at T0, T30, T60, T90, T120, and T180 min.

**Figure 2:**
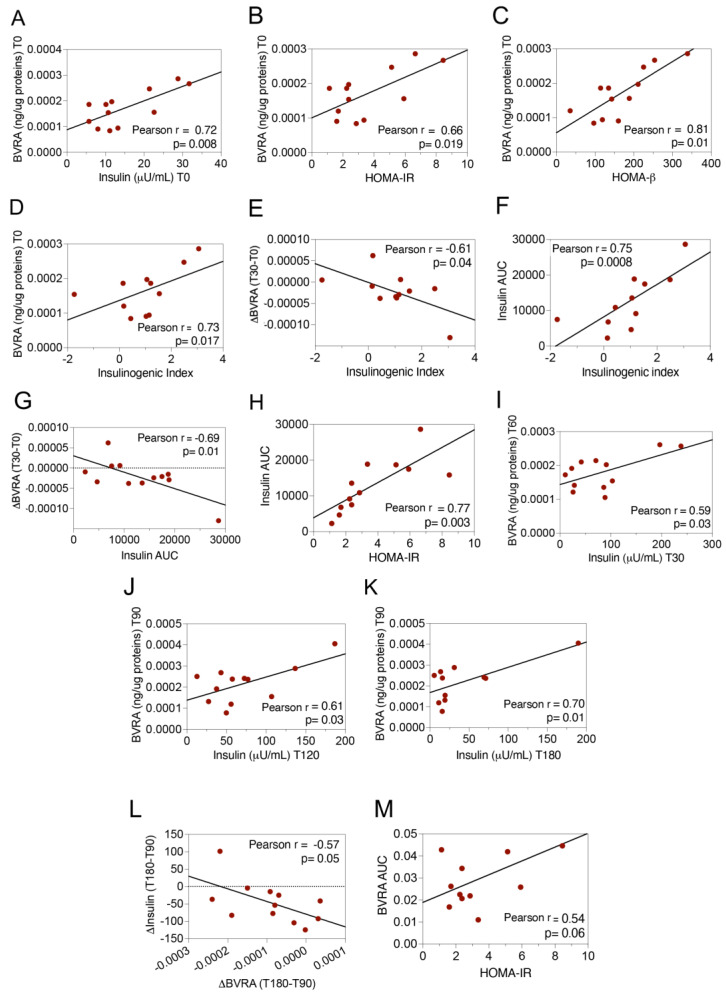
Significant correlations (**A**–**M**) found among BVRA protein levels in PBMCs, circulating insulin levels, and surrogate markers of insulin resistance (Pearson’s coefficient) in our cohort (N = 12).

Our results show a significant overall change of BVRA protein levels across the time (one-way ANOVA F(5,52) = 2.40, *p* < 0.05)) (Figure 1C). Basal BVRA protein levels are positively associated with fasting insulin and indexes of increased insulin resistance and insulin secretion, such as HOMA-IR, HOMA-β, and the insulinogenic index, a validated indicator of acute beta cell response (first-phase insulin secretion: (BI30-FBI0)/(BG30-FBG) (Figure 2A–D). In addition, higher BVRA protein levels at the baseline are predictive of an overall increased BVRA area under the curve (AUC) during the OGTT (0.38 ± 0.014 vs. 0.023 ± 0.008, *p* = 0.04). These findings likely suggest that higher basal BVRA protein levels can be observed in those individuals with lower insulin sensitivity but still normal glycemic control.

During the OGTT, a decrease in BVRA protein levels of about 20% from T0 to T30 that did not reach a statistical significance (Figure 1C) was observed. Despite that, a strong significant negative correlation was found between the rise of circulating insulin levels from T0 to T30 (insulinogenic index) and the respective variation of BVRA protein levels in PBMCs (ΔBVRA (T30–T0)) (Figure 2E), suggesting that the greater first-phase insulin secretion the wider the BVRA protein levels decrease. We have also observed that the insulinogenic index was strongly associated with insulin AUC for each subject (Figure 2F). Hence, the greater the first-phase insulin secretion the lower the insulin sensitivity. Intriguingly, ΔBVRA (T30–T0) is significantly and negatively associated with insulin AUC (Figure 2G). This observation might further support the hypothesis that a greater reduction in BVRA protein levels in response to insulin between T0 and T30 occurs in those subjects with a lower insulin sensitivity. Indeed, insulin AUC strongly and positively correlates with indexes of insulin resistance, e.g., HOMA-IR (Figure 2H). 

**Figure 3:**
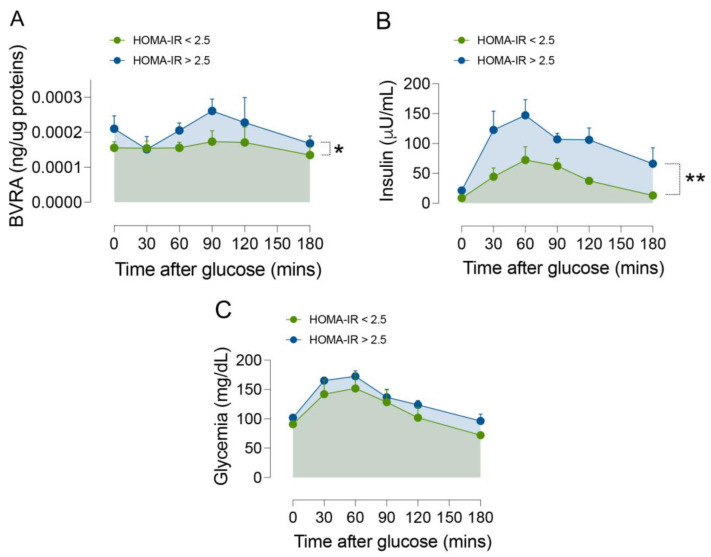
Changes of (**A**) BVRA protein levels (ng/μg of proteins) evaluated in PBMCs along with mean changes of (**B**) insulin (μU/mL) and (**C**) glycemia (mg/dL) circulating levels in the subjects of our cohort categorized according to the HOMA-IR value < 2.5 (*n* = 6) and >2.5 (*n* = 6). Data are presented as mean ± SEM. * *p* < 0.05, ** *p* < 0.01 (paired Student’s *t*-test).

Post hoc tests further show a significant increase in BVRA protein levels of about 43% from T30 to T90 (Figure 1C). The increase in BVRA levels during the OGTT correlates with the increase in insulin under glucose stimulation. Indeed, higher plasma insulin levels at 30 were significantly associated with greater BVRA levels at 60 min (Figure 2I). Similarly, a linear relationship was found between BVRA levels at 90 min and insulin levels at 120 and 180 min (Figure 2J,K). Then, between T90 and T180, a decrease in glycemia, insulin, and BVRA levels to a similar extent was observed at T0 occurs (Figure 1A–C). We observed a significant negative correlation between the change in BVRA levels from T90 to T180 (ΔBVRA (T180–T90)) and the change in insulin levels over the same time period (Δinsulin (T180–T90)) (Figure 2L). This suggests that individuals with reduced insulin sensitivity experience a greater reduction in BVRA levels from T90 to T180, possibly because they require a greater increase in BVRA levels from T30 to T90. These findings lend further support to the hypothesis that individuals with reduced insulin sensitivity may need to achieve higher levels of BVRA to activate the intracellular insulin signaling pathway. Overall levels of BVRA (BVRA AUC) are nearly significantly correlated with HOMA-IR (Figure 2M), strengthening the hypothesis that cells try to foster the activation of the insulin signaling by upregulating BVRA. Indeed, if we categorize the individuals of our cohort based on the HOMA-IR < 2.5 and >2.5, the result is that both BVRA protein level and insulin circulating level changes across the time of the OGTT are significantly different between lower and higher HOMA-IR groups (Figure 3A,B). No significant differences in terms of glycemia were observed (Figure 3C), further supporting the hypothesis that changes of BVRA occur in response to insulin and are greater in those individuals characterized by a lower insulin sensitivity to preserve the euglycemic state. Indeed, we found that a greater insulinogenic index independently predicted increased BVRA AUC during the OGTT after adjustment for age, sex, and BMI at the multivariate regression analysis (β coefficient: 0.012, standardized β: 0.802, *p* = 0.009; Model R: 0.80, R2: 0.644).

### 2.2. Dynamic Changes of BVRA Protein Levels in Response to Insulin in HEK293 Cells

**Figure 4:**
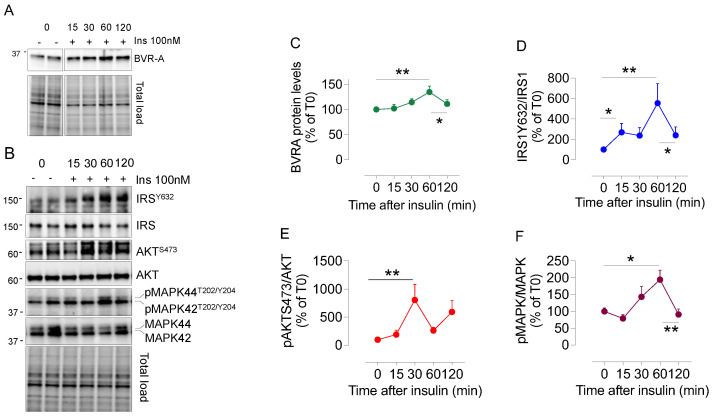
BVRA protein levels along with the activation of main proteins of the insulin signaling pathway were evaluated in HEK293 cells treated with 100 nM insulin for 15, 30, 60, and 120 min. (**A**,**B**) Representative Western blot and total load images and densitometric evaluation of (**C**) BVRA protein levels; (**D**) IRS1 activation evaluated as a ratio between the IRS1 active form (IRS1Y632) and the IRS1 total protein levels (pIRS1Y632/IRS1); (**E**) AKT activation evaluated as a ratio between the AKT active form (AKTS473) and the AKT total protein levels (pAKTS473/AKT); and (**F**) MAPK activation evaluated as a ratio between the MAPK active forms (pMAPK42/44 T202/Y204) and the MAPK total protein levels (pMAPK/MAPK). Data are shown as mean ± SEM (*n* = 4–5 independent cultures/group) One-way ANOVA with Bonferroni post hoc test: * *p* < 0.05, ** *p* < 0.01.

To strengthen the hypothesis that intracellular BVRA protein levels change in response to insulin and that these changes might depend on the different roles BVRA plays within the insulin signaling pathway, e.g., a kinase for IRS1 and a scaffold required for AKT and MAPK activation [1], we evaluated the effect of 100 nM insulin administered to HEK293 cells for 15, 30, 60, and 120 min. The dose of insulin was selected based on our and others’ previous works showing that HEK293 cells are responsive to a such dose [3,28]. The collected results show a significant overall variation across the time of (i) BVRA protein levels (one-way ANOVA F(4,41) = 3.46, *p* < 0.01) (Figure 4A,C); IRS1 activation evaluated as a ratio between the IRS1 active form (IRS1Y632) and the IRS1 total protein levels (pIRS1Y632/IRS1) (one-way ANOVA F(4,25) = 3.76, *p* < 0.05) (Figure 4B,D); AKT activation evaluated as a ratio between the AKT active form (AKTS473) and the AKT total protein levels (pAKTS473/AKT) (one-way ANOVA F(4,33) = 3.45, *p* < 0.05) (Figure 4B,E); and the MAPK activation evaluated as a ratio between the MAPK active forms (pMAPK42/44 T202/Y204) and the MAPK total protein levels (pMAPK/MAPK) (one-way ANOVA F(4,15) = 4.99, *p* < 0.01) (Figure 4B,F). Moreover, post hoc tests reveal significant changes associated with the duration of the treatment that differ among the proteins taken into consideration. In detail, we observed that a significant increase in BVRA protein levels of about 35% can be observed at T60 compared to T0, while BVRA levels reduce to a similar extent in untreated cells at T120 (Figure 4C). IRS1 activation is significantly increased by about 168% at T15 and 450% at T60 compared to T0, while it reduces to 315% at T120 (Figure 4D). In parallel, we observed that AKT activation increases significantly by about 700% at T30 (Figure 4E). Finally, the evaluation of MAPK reveals that these proteins are consistently activated at T60 following insulin administration. MAPK activation increases by 94% at T60 compared to T0, and it reduces to a similar extent in untreated cells at T120 (Figure 4F).

Together, these results further support the notion that the activation of proteins of insulin signaling does not occur simultaneously in response to insulin. Moreover, it appears evident that an increase in BVRA protein levels associates with an increased activation of AKT and MAPK.

## 3. Discussion

This is the first study addressing a key question related to BVRA protein and to its function as a pivotal regulator of the insulin signaling pathway, namely whether BVRA levels change in response to insulin or glucose levels. Our findings bring new information to light, demonstrating that in humans, the levels of intracellular BVRA protein change significantly in response to insulin. This could indicate that evaluating BVRA may be valuable in understanding the molecular processes involved in insulin signaling activation and the development of insulin resistance.

The current report is innovative in that it evaluates the levels of BVRA protein in vivo during an OGTT. During this test, PBMCs are exposed to insulin secreted naturally in response to rising glucose levels. The findings show that BVRA levels change over time, which supports the pleiotropic roles BVRA plays in insulin signaling [1,2]. Intriguingly, the extent of the variations of BVRA protein levels observed in PBMCs during the OGTT in the current study agrees with previous findings from both our group and others, showing that either a decrease or an increase in BVRA protein levels (in a range comprised between 20 and 60%, depending on the cell type/tissue evaluated) leads to an impaired or an increased activation of the insulin signaling, respectively [3,4,6,12,14,25,28,29]. Furthermore, this is in line with previous research indicating that proteins in the signaling pathway do not activate simultaneously in response to insulin [41,42]. This phenomenon was also observed in the cell model used in this study.

**Figure 5:**
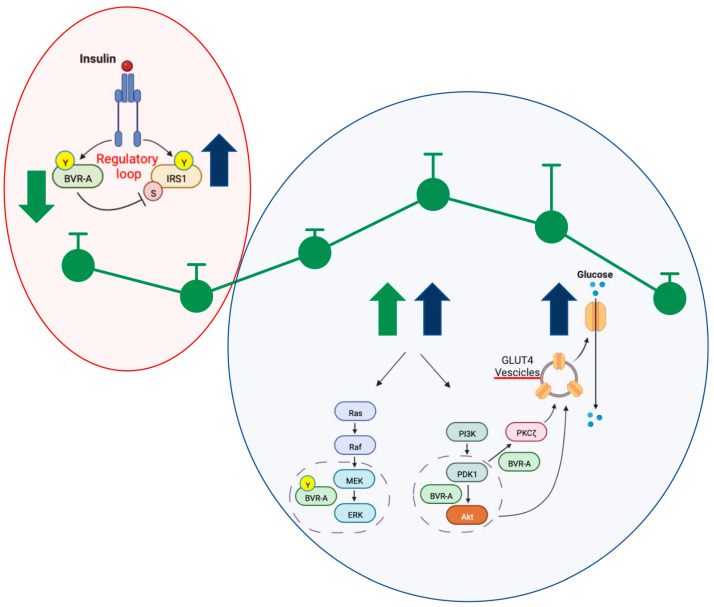
Proposed mechanism through which insulin promotes BVRA protein levels changes depending on the regulatory role for BVRA within the insulin signaling pathway. Upstream in the pathway (red oval)*:* Under physiological conditions, the insulin receptor (IR) activation promotes the phosphorylation of its direct substrate (IRS1) on specific tyrosine (Y) residues. In parallel, IR phosphorylates BVRA on specific Y residues and activates BVRA to function as S/T/Y kinase. Then, as part of a regulatory loop, BVRA phosphorylates IRS1 on inhibitory serine (S) residues (e.g., S307) to avoid IRS1 aberrant activation in response to IR. In subjects with reduced insulin sensitivity, a greater decrease in BVRA protein levels can be observed in response to insulin to favor IRS1 activation. Downstream in the pathway (blue oval): Once activated, IRS1 works as a scaffold protein, driving the activation of the two main arms of the insulin signaling: (1) the Ras/Raf/MAPK pathway (ERK1/2) mainly involved in gene transcription and (2) the PI3K/Akt axis that is critical for glucose uptake as well as for protein and lipid metabolism. Within both axes, BVRA works as a kinase or as a scaffold protein facilitating (1) the ERK1/2 phosphorylation and the subsequent translocation in the nucleus; (2) the PDK1-mediated activation of AKT; and (3) the PDK1-mediated activation of the atypical PKCζ; both these latter events are needed for GLUT4 translocation to the plasma membrane to increase glucose uptake. See text for more details. In subjects with reduced insulin sensitivity, a greater increase in BVRA protein levels can be observed in response to insulin to favor the activation of MAPK and AKT axes. Green arrows refer to BVRA protein levels; blue arrows refer to the molecular events regulated by BVRA.

The finding that BVRA protein levels decrease in response to insulin from T0 to T30 agrees with the regulatory role for BVRA upstream in the signaling pathway, i.e., the IR/BVRA/IRS1 loop [1]. We argue that in the short time, BVRA levels reduce to favor IRS1 activation [1]. Then, BVRA levels increase from T30 to T90 to foster the activation of the intracellular signaling downstream from IRS1, through both its kinase and scaffold functions [1]. Intriguingly, variations of BVRA protein levels across the time of the OGTT are greater in those individuals with lower insulin sensitivity. The significant correlations we found between changes of BVRA levels across the OGTT and measures of insulin sensitivity (insulin T0, insulinogenic index, ΔInsulin (T180–T90), insulin AUC, HOMA-IR, and HOMA-β) agree with this paradigm. Noteworthy, higher BVRA AUC is positively associated with HOMA-IR. Our idea is that, due to the lower insulin sensitivity, the cells try to foster the activation of the signaling by BVRA, to preserve the euglycemic state. In particular, a greater reduction from T0 to T30 would increase IRS1 activation, while the following increase in BVRA protein levels from T30 to T90 would be needed to prompt the activation of both the AKT and MAPK pathways (Figure 5). This hypothesis is corroborated by the results collected in HEK293 cells as well in previous reports [28,41,42] showing that IRS1 activation precedes the activation of either AKT or MAPK in response to insulin and that knocking down BVRA leads to impaired activation of the insulin signaling pathway [3,9,25,28,43]. In particular, we found that HEK cells in which BVRA was silenced with a specific siRNA, do not respond to 100 nM insulin treatment. Indeed, neither AKT/mTOR nor ERK1/2 activation was observed [28]. Rather, the coadministration of a BVRA mimetic peptide with insulin was able to restore the insulin signaling activation in HEK cells lacking BVRA [28]. With a similar approach, but in separate experiments, Maines’s group demonstrated that BVRA is required for the activation of ERK1/2, while silencing BVRA blocked the activation of both ERK and Elk1 transcriptional activity in response to insulin [4,14]. In addition, the same group showed that in the presence of MEK1 and -2 siRNA, there was a significant decrease in the Elk1-dependent activity in HEK stimulated with insulin. The inclusion of BVRA siRNA together with those for MEK further reduced activity, to less than 20% that of the control [4]. Similarly, Maines’s group demonstrated that BVRA is a scaffold protein for AKT and is required to prompt AKT activation in response to insulin [6]. The activation of insulin signaling was recovered by BVRA mimetic peptides in cells lacking BVRA [6,12]. Hence, current and previous findings strongly support a role for BVRA as a critical mediator in insulin signaling activation. It is worth mentioning that in our study, there was no decrease in BVRA protein levels in HEK293 cells treated with insulin. This could be because these cells are responsive to insulin and do not need to foster the activation of IRS1. Conversely, during insulin resistance, IRS1 is inhibited and prevents downstream signaling. In such a scenario, cells might need to enhance IRS1 activation, and one way to do that could be by reducing BVRA levels for a short time following insulin receptor activation (Figure 5). Rather, the observed increase in BVRA protein levels at T60 would be necessary to prompt AKT and MAPK activation both under physiological and pathological conditions [4,6,9]. 

Moreover, the results collected in the current study agree with our previous findings in both obese and T2D subjects in which we observed consistently reduced BVRA levels in PBMC under basal conditions (following overnight fasting), with respect to matched controls [29,30,31]. In obese subjects showing reduced insulin sensitivity with respect to the controls, decreased BVRA levels were associated with a basal hyper-activation of IRS1 and to a less extent of AKT and AS160 [29]—this pathway is known to promote GLUT4 translocation on the plasma membrane to increase glucose uptake, among the others [17]. 

Intriguingly, IRS1 hyperactivation in PBMCs isolated from obese individuals mainly results from reduced Ser307 (inhibitory) phosphorylation [29], the latter being the site for BVRA kinase activity on IRS1 [2,11]. Indeed, through this specific phosphorylation, BVRA avoids the IRS1 aberrant activation in response to the insulin/IR, thus contributing to regulating IRS1 [11]. Hence, reduced BVRA levels in obese subjects likely represent a compensatory mechanism to foster insulin signaling activation in the context of reduced insulin sensitivity. Similar to the results collected in the current study, a positive association was observed between BVRA protein levels and beta cell secretion [44] in obese subjects [29]. On the other hand, we proposed that chronic lower BVRA levels would favor insulin resistance and/or diabetes development (for more details about the mechanisms see [1]). In fact, in subjects with overt T2D, when insulin is not secreted anymore, reduced BVRA levels are associated with a more severe glycometabolic impairment [31]. 

Finally, we acknowledge that our study has a few limitations that need to be addressed in the future. One limitation is the relatively small group of subjects belonging to our cohort. However, despite the sample size, consistent effects with outcome measures suggest this may be a robust effect. Another limitation concerns the evaluation of the insulin signaling activation in PBMCs isolated during the OGTT. Although the results collected in vitro and in animal models either in the current study or in previous works are consistent, the analysis of the IRS1, AKT, and MAPK protein activation in PBMCs isolated during the OGTT might provide additional information to support our hypothesis. Further studies are warranted to fully understand the pathophysiologic processes behind our observations and the possible clinical implications.

## 4. Materials and Methods

### 4.1. Study Population

We recruited twelve consecutive individuals referring to the diabetes and endocrinology outpatient clinics of Sapienza University, Rome, Italy, for metabolic characterization. All the study participants underwent a complete clinical workup including medical history collection, clinical examination, anthropometric measurements, and laboratory tests. Weight, height, and waist circumference were measured, and body mass index was calculated (BMI; weight (kg) × squared height (m^2^)); systemic systolic (SBP) and diastolic (DBP) blood pressure were assessed after 5 min of resting, and mean values of three consecutive assessments were recorded. Overnight fasting blood samples were obtained in all the study participants for routine biochemistry and to collect peripheral blood mononuclear cells (PBMCs). Glycosylated hemoglobin (HbA1c, %—mmol/mol), total cholesterol (mg/dL), HDL-cholesterol (mg/dL), and triglycerides (mg/dL) were measured by standard laboratory methods. LDL-cholesterol (mg/dL) was obtained using the Friedwald formula.

All the subjects underwent OGTT, measuring blood glucose (BG, mg/dL) and insulin values (BI, μU/mL) before and 30, 60, 90, 120, and 180 min after glucose load (75 g). Insulin resistance and secretion were estimated by calculating the main static and OGTT-derived indexes, such as the Homeostasis Model Assessment of Insulin Resistance [HOMA-IR, (FBG * FBI)/22.5] and β cell secretion (HOMA-β (360 * FBI)/(FBG − 63), the Matsuda sensitivity index (ISI, 10,000/(FBG * FBI * BGmean * BImean), the insulinogenic index ((BI30 − FBI0)/(BG30 − FBG), the corrected insulin response (CIR, 100 * BI30/[BG30 * (BG30 − 3.89)], and the disposition index (DI, ISI * CIR). Subjects’ characteristics are shown in Table 1.

### 4.2. Sample Collection

PBMCs were isolated from overnight fasting blood samples at each of the time points of the OGTT (0–30–60–90–120–180 min). ACD-A-anticoagulated blood was centrifuged at 800× *g* for 30 min, and the top layer containing plasma was removed. The remaining blood was diluted with an equal volume of phosphate-buffered saline, pH 7.4 (PBS, (Merck, Darmstadt, Germany), containing 0.05 M ethylenediaminetetraacetic acid (EDTA; Thermo Fisher Scientific Waltham, MA, USA). A total of 12.5 mL of diluted blood was layered over 25 mL of the Ficoll-Paque PLUS (GE Healthcare, Chicago, IL, USA). Gradients were centrifuged at 400× *g* for 30 min at room temperature in a swinging bucket rotor without the brake applied. The PBMC interface was carefully removed by pipetting and washed with PBS-EDTA by centrifugation at 250× *g* for 10 min. PBMC pellets were suspended in ammonium–chloride–potassium lysing buffer (Thermo Fisher Scientific Waltham, MA, USA) and incubated for 10 min at room temperature with gentle mixing to lyse contaminating red blood cells, then washed with PBS-EDTA. PBMCs were cryopreserved in liquid nitrogen in fetal calf serum (FCS; Thermo Fisher Scientific Waltham, MA, USA) containing 10% dimethyl sulfoxide (DMSO; Thermo Fisher Scientific Waltham, MA, USA) and stored until required for downstream analyses. 

### 4.3. Samples Preparation 

Total protein extracts were prepared in RIPA buffer (pH 7.4) containing Tris-HCl (50 mM, pH 7.4) (Merck, Darmstadt, Germany) NaCl (150 mM) (Merck, Darmstadt, Germany), 1% NP-40 (Merck, Darmstadt, Germany), 0.25% sodium deoxycholate (Merck, Darmstadt, Germany), and EDTA (1 mM) (Merck, Darmstadt, Germany), 0.1% SDS (Merck, Darmstadt, Germany), supplemented with proteases inhibitors [(phenylmethylsulfonyl fluoride (PMSF, 1 mM), sodium fluoride (NaF, 1 mM), and sodium orthovanadate (Na_3_VO_4_, 1 mM) (all from Merck, Darmstadt, Germany)]. PBMCs were homogenized by 20 passes with a Wheaton tissue homogenizer and then clarified by centrifugation for 1 h at 16,000× *g*, 4 °C. The supernatant was then extracted to determine the total protein concentration by the Bradford assay (Pierce, Rockford, IL, USA).

### 4.4. BVRA ELISA

BVRA protein levels in PBMCs were evaluated by an ELISA kit (Cat#EKU02723, Biomatik, Kitchener, Ontario, Canada)according to the manufacturer’s instructions. The amount of BVRA was then normalized for total proteins of PBMC samples loaded into the ELISA kit and expressed as ng/μg proteins.

### 4.5. HEK293 Cells

Human Embryonic Kidney (HEK) 293 cells were grown in Dulbecco’s modified Eagle’s medium (DMEM, GIBCO, Gaithersburg, MD, U.S.A) supplemented with 10% fetal bovine serum (FBS), 2 mM l-glutamine, penicillin (20 units/mL), and streptomycin (20 mg/mL) (GIBCO, Gaithersburg, MD, U.S.A.). Cells were maintained at 37 °C in a saturated humidity atmosphere containing 95% air and 5% CO_2_. To test the effect of insulin on BVRA protein levels, cells were seeded at 75 × 10^3^/cm^2^ in 24-well culture dishes with 1% FBS. After 24 h, cells were treated with 100 nM insulin (Humulin R, Ely-Lilly, Indianapolis, IN, USA) for 15, 30, 60, and 120 min as previously described [3,28]. At the end of each treatment, the medium was removed, cells were washed twice with PBS and collected, and proteins were extracted as described above.

### 4.6. Western Blot

Fifteen μg of protein was resolved via SDS-PAGE using Criterion™ TGX Stain-Free™ precast gel (Bio-Rad Laboratories, Hercules, CA, USA) in a Criterion large format electrophoresis cell (Bio-Rad Laboratories, Hercules, CA, USA) in Tris/Glycine/SDS (TGS) Running Buffer (Bio-Rad Laboratories, Hercules, CA, USA). Immediately after electrophoresis, the gel was placed on a Chemi/UV/Stain-Free tray and then placed into a ChemiDoc MP imaging System (Bio-Rad Laboratories, Hercules, CA, USA) and UV-activated based on the appropriate settings with Image Lab Software (Bio-Rad Laboratories, Hercules, CA, USA) to collect total protein load image. Following electrophoresis and gel imaging, the proteins were transferred onto a nitrocellulose membrane by Trans-Blot Turbo Transfer System (Bio-Rad Laboratories, Hercules, CA, USA). To prove the transfer, the blot was imaged by the ChemiDoc MP imaging system using the Stain-Free Blot settings. The nitrocellulose membrane was blocked using 3% BSA (SERVA Electrophoresis GmbH, Heidelberg, Germany) in 1X Tris Buffer Saline (TBS) containing 0.01% Tween20 (Sigma-Aldrich, St. Louis, MO, USA) and incubated overnight at 4 °C with the following primary antibodies: anti-BVRA (1:1000, Merck, Darmstadt, Germany, #B8437); anti-IRS1 (1:1000, Cell Signaling Technology, Danvers, MA, USA, #2382); anti-phospho-IRS1Y632 (1:500, Santa Cruz, Dallas, TX, USA, SC17196); anti-AKT (1:1000, ECM Bioscience, Aurora, CO, USA, #AM-1011); anti-phospho-AKTS473 (1:1000, Thermo Fisher Scientific Waltham, MA, USA, #44621); anti-MAPK (1:1000, Cell Signaling Technology, Danvers, MA, USA, #9012S); and anti-phospho-MAPK (1:1000, Cell Signaling Technology, Danvers, MA, USA, #9106S). The day after, all membranes were washed with 1X TBS containing 0.01% Tween20 (Merck, Darmstadt, Germany) and incubated at room temperature for 1 h with an anti-rabbit (1:1000; Bio-Rad Laboratories, Hercules, CA, USA) secondary antibody conjugated with horseradish peroxidase. Membranes were developed with Clarity enhanced chemiluminescence (ECL) substrate (Bio-Rad Laboratories, Hercules, CA, USA) and then acquired with ChemiDoc MP (Bio-Rad, Hercules, CA, USA) and analyzed using Image Lab 6.1 software (Bio-Rad, Hercules, CA, USA) that allows the normalization of a specific protein signal by the total protein load. Total protein staining measures the aggregate protein signal (sum) in each lane and eliminates the error that can be introduced by a single internal control protein. Total protein staining is a reliable and widely applicable strategy for quantitative immunoblotting. It directly monitors and compares the aggregate amount of sample protein in each lane, rather than using an internal reference protein as a surrogate marker of sample concentration. This direct, straightforward approach to protein quantification may increase the accuracy of normalization. Total load can be detected by taking advantage of the stain-free technology (Bio-Rad, Hercules, CA, USA). Stain-free imaging technology utilizes a proprietary trihalo compound to enhance natural protein fluorescence by covalently binding to tryptophan residues with a brief UV activation. Images of the gel or membrane after transfer can easily be captured multiple times without staining and destaining steps. 

### 4.7. Statistics

All data are presented as mean  ±  SEM or standard deviations (SD) of *n* independent samples per group. Human and cellular data were evaluated using one-way analysis of variance (ANOVA) with Bonferroni’s Multiple Comparisons. Association between BVRA protein levels and metabolic parameters, i.e., BG and BI, as well as static and dynamic indexes of insulin resistance and secretion, were tested by Pearson’s coefficient. To identify independent predictors of BVRA dynamic changes during the OGTT, we built multivariate regression models starting from parameters significantly associated with the bivariate analyses and forcing for sex and age. Results from the final stepwise model are reported in the text. R^2^ was calculated as a goodness-of-fit measure for the regression model; both R and R^2^ were reported. All statistical tests were two-tailed, and the level of significance was set at 0.05. Statistical analyses were performed with SPSS for MacOs (version 27, SPSS Inc., Chicago, IL, USA).

## Figures and Tables

**Table 1:** Clinical characteristics of the study population.

Age	48.5 ± 15.8
Sex (%M)	16.7%
BMI (kg/m^2^)	35.2 ± 9.5
Waist circumference (cm)	123 ± 13.9
SBP (mmHg)	121.2 ± 13.8
DBP (mmHg)	73.7 ± 6
FBG (mg/dL)	96.5 ± 12.4
FBI (IU/mL)	15 ± 8.8
HbA1c (mmol/mol; %)	37 ± 4, 5.5 ± 0.4%
Total cholesterol (mg/dL)	186.7 ± 32.2
HDL cholesterol (mg/dL)	56.6 ± 17.9
LDL cholesterol (mg/dL)	105.9 ± 23
Triglycerides (mg/dL)	119.7 ± 89.8
HOMA-IR	3.65 ± 2.3
HOMA-β	168.3 ± 80.8
Insulinogenic index	1.23 ± 0.94
Matsuda Index	3.87 ± 3.32
CIR	0.81 ± 0.62
Disposition Index	3.21 ± 2.2

Data are shown as mean ± standard deviation or percentage. Abbreviations: BMI—body mass index, SBP—systolic blood pressure, DBP—diastolic blood pressure, FBG—fasting blood glucose, FBI—fasting blood insulin, HDL—high density lipoprotein, LDL—low density lipoprotein, HOMA-IR—Homeostasis Model Assessment of Insulin Resistance, HOMA-β—Homeostasis Model Assessment of beta cell secretion, CIR—corrected insulin response.

## Data Availability

Additional datasets that support the findings of this study are available from the corresponding author upon request.
